# *S100B* single nucleotide polymorphisms exhibit sex-specific associations with chronic pain in sickle cell disease in a largely African-American cohort

**DOI:** 10.1371/journal.pone.0232721

**Published:** 2020-05-07

**Authors:** Ellie H. Jhun, Nilanjana Sadhu, Ying He, Yingwei Yao, Diana J. Wilkie, Robert E. Molokie, Zaijie Jim Wang

**Affiliations:** 1 Department of Pharmaceutical Sciences, University of Illinois College of Pharmacy, Chicago, Illinois, United States of America; 2 Comprehensive Sickle Cell Center, University of Illinois at Chicago, Chicago, Illinois, United States of America; 3 Department of Biobehavioral Nursing Science, University of Florida College of Nursing, Gainesville, Florida, United States of America; 4 Jesse Brown Veteran’s Administration Medical Center, Chicago, Illinois, United States of America; 5 Division of Hematology/Oncology, University of Illinois at Chicago College of Medicine, Chicago, Illinois, United States of America; 6 Department of Neurology & Rehabilitation, University of Illinois at Chicago College of Medicine, Chicago, Illinois, United States of America; Universidade Nova de Lisboa Instituto de Higiene e Medicina Tropical, PORTUGAL

## Abstract

**Background:**

Pain in sickle cell disease (SCD) is severe and multifaceted resulting in significant differences in its frequency and intensity among individuals. In this study, we examined the influence of *S100B* gene single nucleotide polymorphisms (SNP) on acute and chronic pain variability in SCD.

**Methods:**

Composite pain index (CPI) scores captured chronic pain. Painful crisis related emergency care utilization recorded acute pain incidence. Genotyping was performed using *MassARRAY iPLEX* platform.

**Results:**

Regression analysis revealed associations of increased CPI with rs9722 A allele in additive (p = 0.005) and dominant (p = 0.005) models. Rs1051169 G allele on the other hand was associated with decreased CPI in additive (p = 0.001), and dominant (p = 0.005) models. Sex-specific analysis found that these associations were significant in females but not males in this cohort. Linkage analysis identified two haploblocks. Block 1 (rs9983698-rs9722) haplotype T-A was associated with increased CPI (p = 0.002) while block 2 (rs1051169-rs11911834) haplotype G-G was associated with decreased CPI (p = 0.001). Both haplotypic associations were only significant in females. No association of *S100B* SNPs with utilization reached statistical significance.

**Conclusions:**

*S100B* SNPs and haplotypes are associated with chronic pain in female, but not male, patients with SCD, implicating a potential role of *S100B* polymorphism in SCD pain heterogeneity in a sex-dependent manner.

## Introduction

Pain in sickle cell disease (SCD) is a significant problem that contributes to considerable morbidity and mortality as well as health care cost [1, 2]. Both acute and chronic pain in SCD are highly variable in frequency and severity, and the underlying cause of individual differences in SCD pain remains largely unknown [3–8]. To understand the individual differences in pain, we took a genetic approach to identify genetic polymorphism that may contribute to SCD pain heterogeneity.

The *S100B* gene codes for a calcium binding protein of the S-100 protein family and is located on chromosome 21q22.3 near risk regions for Alzheimer’s disease, bipolar affective disorder, and down syndrome [9, 10]. It is abundant in the central nervous system being predominantly expressed in and secreted by astrocytes [11, 12]. In its dimeric form the 21 kDa protein acts as a paracrine and autocrine factor involved in intracellular calcium homeostasis, cell proliferation and differentiation, and signal transduction in the central nervous system [10]. S100B has been extensively researched for its role in brain injury where elevated levels are indicative of the severity of trauma [13]. Increased S100B production in activated astrocytes has been shown to play a role in neuroinflammation and pain signaling.

In a study published earlier last year it was found that the severity of allodynia in an incomplete spinal cord injury model significantly correlated with increase in S100B expression at the site of injury. Suppression of astrocytic activation and inhibition of S100B expression using a pharmacological agent resulted in reduced neuropathic pain [14]. Other models of neuropathic pain and chronic inflammation have also reported increased S100B expression in astrocytes. For example, it was found that the spinal dorsal horn and dorsal root ganglion of complete Freund’s adjuvant (CFA)-treated mice showed increased expression of S100B and upon deletion of *TRPV1*—a receptor critical in pain signaling—this effect was reversed [15]. In a formalin-induced conditioned place avoidance model in rats, expression of astrocytic marker S100B along with proinflammatory cytokines TNF-α and IL-1β were increased in the anterior cingulate cortex [16]. Similar findings have been reported in animal models of tooth injury [17], CFA-induced chronic inflammation [18], and peripheral neuropathic pain [19]. In fact, *S100B* knock-out resulted in decreased mechanical allodynia and *S100B* over-expression caused increased mechanical allodynia in mice post nerve transection as compared to their wild-type counterparts [19]. In addition to pain, elevated levels of *S100B* have also been implicated in avascular necrosis in sickle cell [20, 21]. Avascular necrosis is a complication of sickle cell caused by repeated episodes of vaso-occlusion. It can affect the function of multiple joints in the body and often results in substantial pain [22].

Therefore, *S100B* serves as a potential candidate gene that may influence pain heterogeneity in SCD. In this study, four *S100B* single nucleotide polymorphisms (SNPs) were examined in our SCD cohort, including rs1051169, a synonymous SNP on exon 2, an intronic rs11911834 SNP, rs9983698 and rs9722 in the 3’untranslated region. We determined the genotype and allele frequencies of these SNPs, their associations with acute and chronic pain in SCD patients, and potential sex-specific differences.

## Methods

### Subject recruitment

This study was approved by and conducted in accordance with the ethical standards of the Institutional Review Board of the University of Illinois at Chicago (UIC). Subjects provided written informed consent before blood or buccal swab samples and phenotype data were collected. For participants under the age of 18, parental consent and child assent were obtained. Recruitment of subjects occurred during routine outpatient clinic visits at the University of Illinois Hospital and Health Sciences System (UI) in Chicago, IL and its Sickle Cell Clinic. Study eligibility, pain phenotype assessment and genotyping methods are detailed in some of our previous studies [6, 23–25].

### Pain assessment

Briefly, the Composite Pain Index (CPI) value represents the multidimensional attributes of SCD pain at baseline. The CPI score was calculated by proportionally converting averaged raw scores to a 0 to 100 scale. Baseline pain score included the number of pain sites, average of pain intensity (current, least, and worst intensity in past 24 hours), pain quality, and pain pattern [6, 26–28]. These pain data were collected by a self-administered electronic touch screen format of the 1970 McGill Pain Questionnaire [8, 26, 29] which has been validated for SCD [6].

On the other hand, utilization, which is defined as the number of admissions to the emergency department and acute care center as the result of sickle cell pain crises, was used to measure acute pain in this study. The number of utilizations was recorded for 12 months after subjects completed the baseline pain assessment by medical record review (UI utilization) or by biweekly telephone calls (non-UI utilization).

### DNA isolation and genotyping

For candidate SNP genotyping, DNA from blood and buccal swab samples were initially extracted as described in prior studies [23, 30, 31]. The QuickGene DNA whole blood extraction kit (AutoGen, MA, USA) or a salting-out approach adopted from Miller et al. was used for DNA isolation from blood [32]. Whereas, a phenol/chloroform method adopted from Vandenbergh et al. was used for DNA isolation from buccal swab [33]. Extracted DNA samples were then aliquoted and stored at -80°C. Genotyping was performed on the MassARRAY iPLEX Platform (Sequenom, San Diego, CA) by the University of Illinois at Chicago Research Resource Center. The Assay Design Suite software was used to design extension primers for each SNP to complement upstream of the polymorphic site and generate optimized multiplexed assays. PCR amplification and multiplexed extension reactions were followed by subsequent measurement by MALDI-TOF mass spectrometry [34]. The iPLEX extension primers of the four SNPs were as follows: AGATGCGCTCTTTTTATTGAA for rs9983698, CCTTTCGTGTAACAGAGA for rs9722, GGAATCACAAGCTGAAGAAATCCGAACT for rs1051169, and TTTTATCATAATCCTTCCACC for rs11911834. SNPs were selected based on extensive literature search. Since *S100B* polymorphisms have not been previously studied in the context of pain, we included SNPs that have been associated with comorbid neurological disorders in prior studies [9, 35–38]. Genotyping success rate of SNPs included in this study analyses was >90% and SNPs were in Hardy-Weinberg equilibrium as determined by a χ^2^ goodness-of-fit test.

### Statistical analysis

136 subjects with complete phenotype information were included in the study. Genotype call rate of included SNPs were >90%. Data are expressed as Mean ± Standard Deviation (SD). Multiple linear regression analyses were performed to examine SNP effects on CPI adjusted for age, sex, ethnicity, and sickle cell type [39, 40]. For utilization, since the variances were higher than the mean utilization within the same genotype group, we used negative binomial regression to examine SNP effects on utilization adjusted for the same covariates [41]. We used additive and dominant genetic models for association analyses in overall cohort as well as for sex-specific analyses [40]. Subjects with missing genotype data for a specific SNP were excluded from that association analysis. R (version 3.4.0) was used to perform regression (R package ‘MASS’) and haplotype (R package ‘hapassoc’) analyses. P-values were adjusted for multiplicity using the Benjamini & Hochberg method [42]. Linkage disequilibrium (LD) plot was generated in Haploview version 4.2 (Broad Institute, Cambridge, MA, USA) [43] using the standard color scheme displaying the linkage disequilibrium coefficient (D’) values. Haploblocks were determined by the (default) D’ confidence interval method [44]. For expression quantitative loci (eQTL) analysis, we used the Genotype-Tissue Expression (GTEx) online eQTL calculator (version November 2019) to obtain genotype data of SNPs rs9722 and rs1051169, and the corresponding normalized S100B gene expression in whole blood. The GTEx project characterizes eQTLs across multiple tissues, whole blood being one of them. For whole blood, the database had a total of 670 samples with genotype data and gene expression levels.

## Results

### Subject characteristics

One hundred and thirty-six subjects with genotype and phenotype data were included in this association study. The age range of our subjects was 15 to 70 years with an average of 34.0 ± 11.7 years (Table 1). Our cohort was predominantly self-reported African American (97%), with 3 Hispanics and 1 Caucasian. The majority of our subjects had sickle cell anemia (77%), which is the homozygous hemoglobin S genotype. Female subjects predominated in our study (65%), although our recruitment had no gender preference. SCD is not sex-linked, although gender differences in SCD have previously been observed. Males showed higher mortality than females [1], and males were more frequently admitted for pain crises than females [45].

**Table 1:** Subject summary, n = 136.

Age (years)	Mean ± SD^a^	34.0 ± 11.7
	Minimum	15
	Maximum	70
Sex, n (%)	Male	47 (35)
	Female	89 (65)
Sickle Cell Type^†^, n (%)	SCD-SS	105 (77)
	SCD-SC	15 (11)
	SCD-Sβ^+^	8 (6)
	SCD-Sβ°	7 (5)
	SCD-Sα	1 (1)
Ethnicity, n (%)	African American	132 (97)
	Hispanic	3 (2)
	Caucasian	1 (1)

^a^Standard Deviation.

^†^Sickle cell types: SCD-SS (sickle cell disease-homozygous hemoglobin S, sickle cell anemia), SCD-SC (sickle cell disease-sickle hemoglobin C), SCD-Sβ^+^ (sickle cell disease-sickle β^+^ thalassemia), SCD-Sβ° (sickle cell disease-sickle β° thalassemia), SCD-Sα (sickle cell disease-sickle α thalassemia). Ethnicity is self-reported during the pain assessment

### Phenotype and genotype characteristics

Pain phenotype summary is given in Table 2. Mean CPI was 40.4 ± 13.5; however, the score ranged from 14.8 to 86.5 on a scale of 0 to 100, reaffirming pain heterogeneity in SCD. Utilization was also highly variable with a range from 0 to 38 and a mean of 4.4 ± 5.2. Independent t-test revealed that pain scores were not significantly different between males and females for either CPI (p = 0.18) or utilization scores (p = 0.23). The number of subjects within each utilization group is also summarized in Table 2 and is categorized as described in Methods.

**Table 2:** Phenotype summary, n = 136.

CPI^a^	Mean ± SD^b^
Total	40.4 ± 13.5
Male	38.2 ± 13.6
Female	41.5 ± 13.4
Utilization	Mean ± SD
Total	4.4 ± 5.2
Male	3.7 ± 4.6
Female	4.8 ± 5.5
Utilization Groups^c^	n (%)
Zero (0)	19 (14)
Low (1–3)	60 (44)
High (4–38)	57 (42)

^a^Composite Pain Index.

^b^Standard Deviation.

^c^Utilization groups are categorized according to a previous study in which CPI was associated with these utilization groups

Genotype and allele frequencies are listed in Table 3. No significant deviation from Hardy-Weinberg equilibrium was observed for all 4 SNPs (p>0.05). Two 3’-UTR SNPs (rs9983698, 9722), a synonymous SNP (rs1051169), and an intronic SNP (rs11911834) were included in this study. Major alleles are listed first as all analyses were performed with the major allele/genotype as reference. *S100B* gene is located on chromosome 21q22.3. Genotyping call rate was >90% for all four SNPs included in the study. After accounting for availability of genotype and phenotype information, the total number of subjects for each SNP that were included in regression analyses is noted in Table 3.

**Table 3:** Genotype frequencies.

dbSNP ID	Chr Position	Gene Location	Major Homozygous n (%)	Heterozygous n (%)	Minor Homozygous n (%)	Total (n)	Genotyping call rate (%)
9983698 [C>T]	46598630	3’UTR	84 (64)	41 (31)	7 (5)	132	97%
9722 [G>A]	46599326	3’UTR	44 (35)	54 (43)	27 (22)	125	92%
1051169 [C>G]	46602317	Exon 2	45 (34)	66 (50)	21 (16)	132	97%
11911834 [G>T]	46602608	Intron	83 (61)	45 (33)	7 (5)	135	99%

dbSNP IDs and chromosome positions (GRCh38) are from the National Center for Biotechnology Information database (NCBI). 3’UTR: 3’ untranslated region.

### CPI single SNP regression analysis

Single SNP regression analyses (Table 4) revealed that the A allele of rs9722 was associated with a CPI increase of 5.24 (adjusted p = 0.005) and the AA and GA genotypes combined was associated with a CPI increase of 8.08 (adjusted p = 0.005). For rs1051169, a decrease in CPI was associated with the G allele in the additive model (B = -6.95, adjusted p = 0.001), as well as with the GG and CG genotypes combined as seen in the dominant model (B = -7.95, adjusted p = 0.005).

**Table 4:** Single SNP regression analysis of CPI.

dbSNP ID	Model	B (95% CI)^a^	p-value	Adjusted p-value
9983698	Additive	4.19 (0.20, 8.17)	0.040	0.053
	Dominant	5.44 (0.53, 10.34)	0.030	0.051
9722	Additive	5.24 (1.96, 8.52)	0.002^b^	0.005^b^
	Dominant	8.08 (3.02, 13.14)	0.002^b^	0.005^b^
1051169	Additive	-6.95 (-10.33, -3.57)	0.0001^b^	0.001^b^
	Dominant	-7.95 (-12.82, -3.08)	0.002^b^	0.005^b^
11911834	Additive	4.16 (0.25, 8.07)	0.037	0.053
	Dominant	5.37 (0.61, 10.13)	0.027	0.051

Regression models are adjusted for age, sex, ethnicity, and sickle cell type. Benjamini & Hochberg method used for multiple correction of p-value. The major alleles are the reference genotypes in all analyses.

^a^Unstandardized regression coefficient and 95% confidence interval.

^b^Significant p-values (adjusted p-values ≤0.05)

We also found that the T allele of rs9983698 showed trend for association with higher CPI (B = 4.19, p = 0.040, adjusted p = 0.053) in the additive model and the dominant model (B = 5.44, p = 0.030, adjusted p = 0.051). The T allele for rs11911834 exhibited trend for associating with an increase in CPI by 4.16 (p = 0.037, adjusted p = 0.053) and TT and GT genotype combined with an increase of 5.37 (p = 0.027, adjusted p = 0.051).

### Utilization single SNP regression analysis

Regression analyses using SNP effects on utilization data showed association only for rs1051169 (Table 5). The G allele of rs1051169 was associated with decreased utilization in the additive (IRR = 0.75, p = 0.031) model at a nominal level. However, adjusted p-value did not reach statistical significance. None of the models (additive and dominant) were significant for rs9983698, rs9722 and rs11911834.

**Table 5:** Single SNP regression analysis of utilization.

dbSNP ID	Model	IRR (95% CI)^a^	p-value	Adjusted p-value
9983698	Additive	0.86 (0.64, 1.15)	0.301	0.401
	Dominant	0.78 (0.54, 1.13)	0.181	0.375
9722	Additive	1.17 (0.92, 1.49)	0.207	0.375
	Dominant	1.26 (0.86, 1.83)	0.226	0.375
1051169	Additive	0.75 (0.58, 0.98)	0.031	0.204
	Dominant	0.76 (0.53, 1.10)	0.144	0.375
11911834	Additive	0.84 (0.64, 1.12)	0.250	0.375
	Dominant	0.76 (0.54, 1.09)	0.137	0.375

Regression models are adjusted for age, sex, ethnicity, and sickle cell type. Benjamini & Hochberg method used for multiple correction of p-value. The major alleles are the reference genotypes in all analyses.

^a^Incident rate ratio and 95% confidence interval

### Sex-specific association of SNPs with CPI

Upon further analysis of data by performing regression analyses separately in males and females, rs1051169 was found to exhibit strong sex-specific association with CPI only in females (Table 6). The G allele of rs1051169 was found to be associated with decrease CPI in females (additive model adjusted p = 0.001, dominant model adjusted p = 0.006) but not in males. A similar sex-specific association was observed for rs9722 (Table 6), where the A allele was associated significantly with increased CPI in females (adjusted p = 0.042) but not in males in the additive model. No sex-specific association with CPI was observed for the other SNPs. Association analyses with utilization did not yield any significant association for the four SNPs in either male or female cohort.

**Table 6:** Sex specific single SNP regression analysis of CPI.

dbSNP ID	Sex	Total subjects	B^a^ (p-value)	
			Additive	Dominant
9722	M	44	4.38 (0.440)	10.73 (0.324)
	F	81	5.14 (0.042)^b^	6.69 (0.084)
1051169	M	46	-2.95 (0.605)	-3.32 (0.608)
	F	86	-8.60 (0.001)^b^	-10.27 (0.006)^b^

Regression models are adjusted for age, ethnicity, and sickle cell type. Benjamini & Hochberg method used for multiple correction of p-value. The major alleles are the reference genotypes in all analyses.

^a^Unstandardized regression coefficient.

^**b**^Significant p-values (adjusted p-values ≤0.05)

### Linkage disequilibrium and haplotype analysis

**Fig 1:**
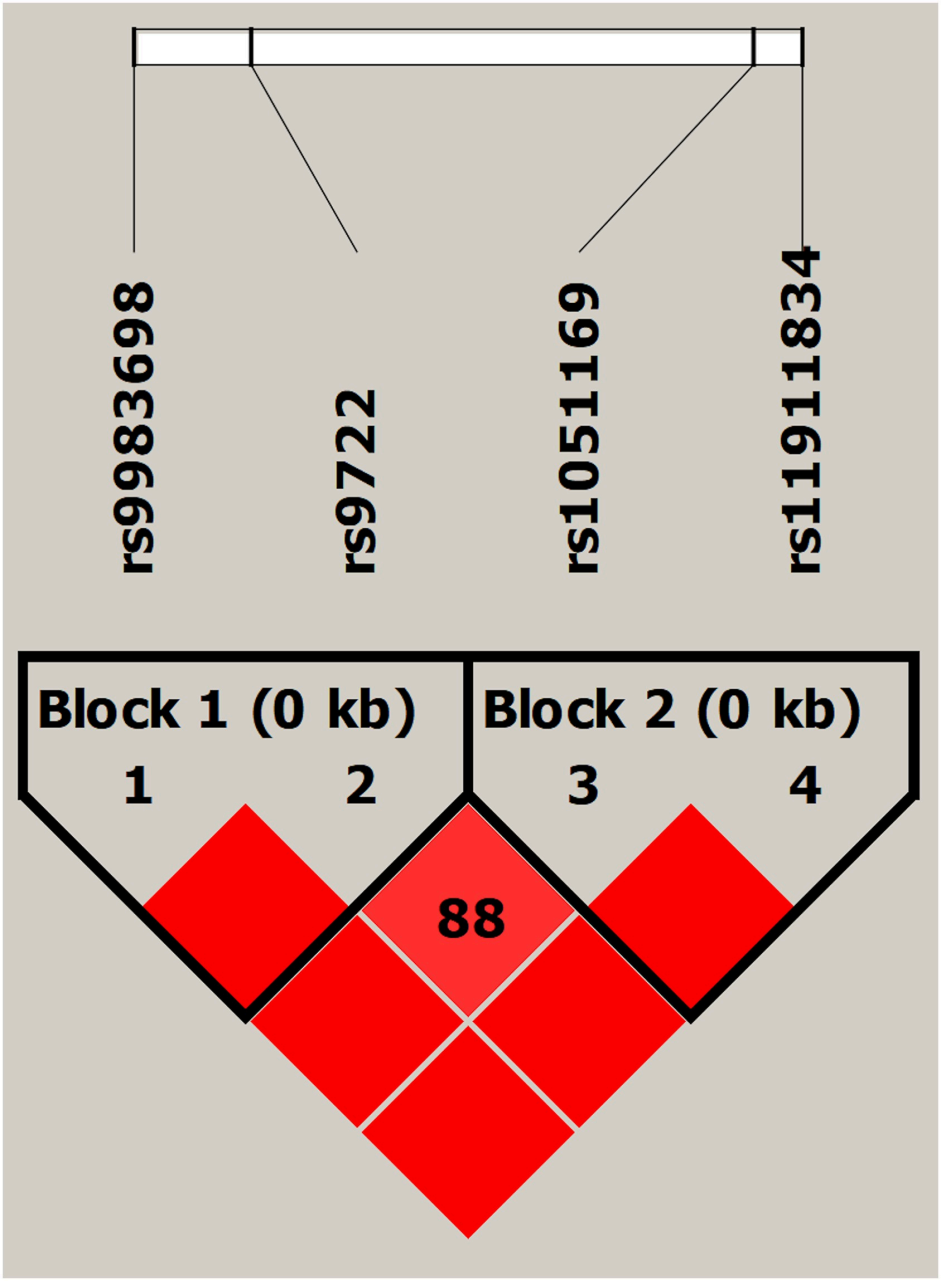
Linkage disequilibrium plot for S100B SNPs.

We observed several significant SNPs from our CPI association data; therefore, we generated a LD plot of the 4 SNPs using Haploview 4.2 (Fig 1) for our sickle cell cohort as multiple significant results from the same gene may imply linkage disequilibrium between the SNPs. Two haplotype blocks were formed (block 1: rs9983698-rs9722; block 2: rs1051169-rs11911834). Haplotype blocks did not change when four non-African American subjects were excluded.

Plot uses the Haploview standard color scheme with D’ values displayed. Red = high D’ and high LOD, white = low D’ and low LOD, blue = high D’ and low LOD, shades of pink/red = low D’ and high LOD; where D’ is the linkage disequilibrium coefficient and LOD is the logarithm of odds score.

Association analyses (Table 7) revealed that haplotype T-A of block 1 (rs9983698-rs9722) was significantly associated with higher CPI (B = 5.99, p = 0.002) compared to the reference haplotype C-G. This is in agreement with single SNP analyses where the risk allele A of rs9722 was associated with an increase in CPI and T allele of rs9983698 showed a similar trend as compared to their respective reference alleles.

**Table 7:** Haplotype frequencies and CPI association analyses.

Haplotype Blocks ^a^	Haplotype	Frequency (%)	Regression coefficient	p-value
Block 1 rs9983698 [C>T]—rs9722 [G>A]	CG	57	*reference haplotype*	
	CA	22	3.46	0.070
	TA	21	5.99	0.002^b^
Block 2 rs1051169 [C>G]—rs11911834 [G>T]	CG	37	*reference haplotype*	
	CT	22	1.99	0.333
	GG	41	-5.84	0.001^b^

^a^Block numbers are from Fig 1 LD plot. Association analyses were performed considering covariates (age, sex, ethnicity, sickle cell type).

^**b**^Significant p-values (p ≤0.05)

Additionally, we performed haplotype analysis separately in males and females to ascertain if the sex specific associations observed in the single SNP studies were reflected in the haplotypes (Table 8). We found that the association of haplotype T-A with higher CPI reached statistical significance in females (p = 0.010) but not in males (p = 0.117).

**Table 8:** Sex specific haplotype analysis of block 1^a^ rs9983698 [C>T]—rs9722 [G>A].

Sex	Haplotype	Frequency (%)	Regression coefficient	p-value
Males (n = 47)	CG	55.5	*reference haplotype*	
	CA	22.1	1.10	0.751
	TA	22.4	5.45	0.117
Females (n = 89)	CG	57.5	*reference haplotype*	
	CA	22.5	4.02	0.085
	TA	20.5	6.28	0.010^b^

^a^Block number is from LD plot (Fig 1). Association analyses were performed considering covariates (age, ethnicity, sickle cell type).

^**b**^Significant p-values (p ≤0.05)

In block 2 (rs1051169-rs11911834), the G-G haplotype was significantly associated with decreased CPI (B = -5.84, p = 0.001) as compared to reference haplotype C-G. Block 2 associations are also in agreement with single SNP analyses where G allele of rs1051169 associated significantly with a decrease in CPI significantly. It may be noted here that the contribution of rs11911834 is not significant and is overpowered by the strong association of rs1051169 with CPI. Furthermore, we compared males versus females and found that this haplotype association was sex specific as well (Table 9). The G-G haplotype associated with decreased CPI only in females (p = 0.004).

**Table 9:** Sex specific haplotype analysis of block 2^a^ rs1051169 [C>G]—rs11911834 [G>T].

Sex	Haplotype	Frequency (%)	Regression coefficient	p-value
Males (n = 47)	CG	35.6	*reference haplotype*	
	CT	23.4	4.21	0.247
	GG	41.0	-1.32	0.675
Females (n = 89)	CG	38.2	*reference haplotype*	
	CT	21.0	1.30	0.597
	GG	40.9	-7.72	0.004^b^

^a^Block number is from LD plot (Fig 1). Association analyses in were performed considering covariates (age, ethnicity, sickle cell type).

^**b**^Significant p-values (p ≤0.05)

**Fig 2:**
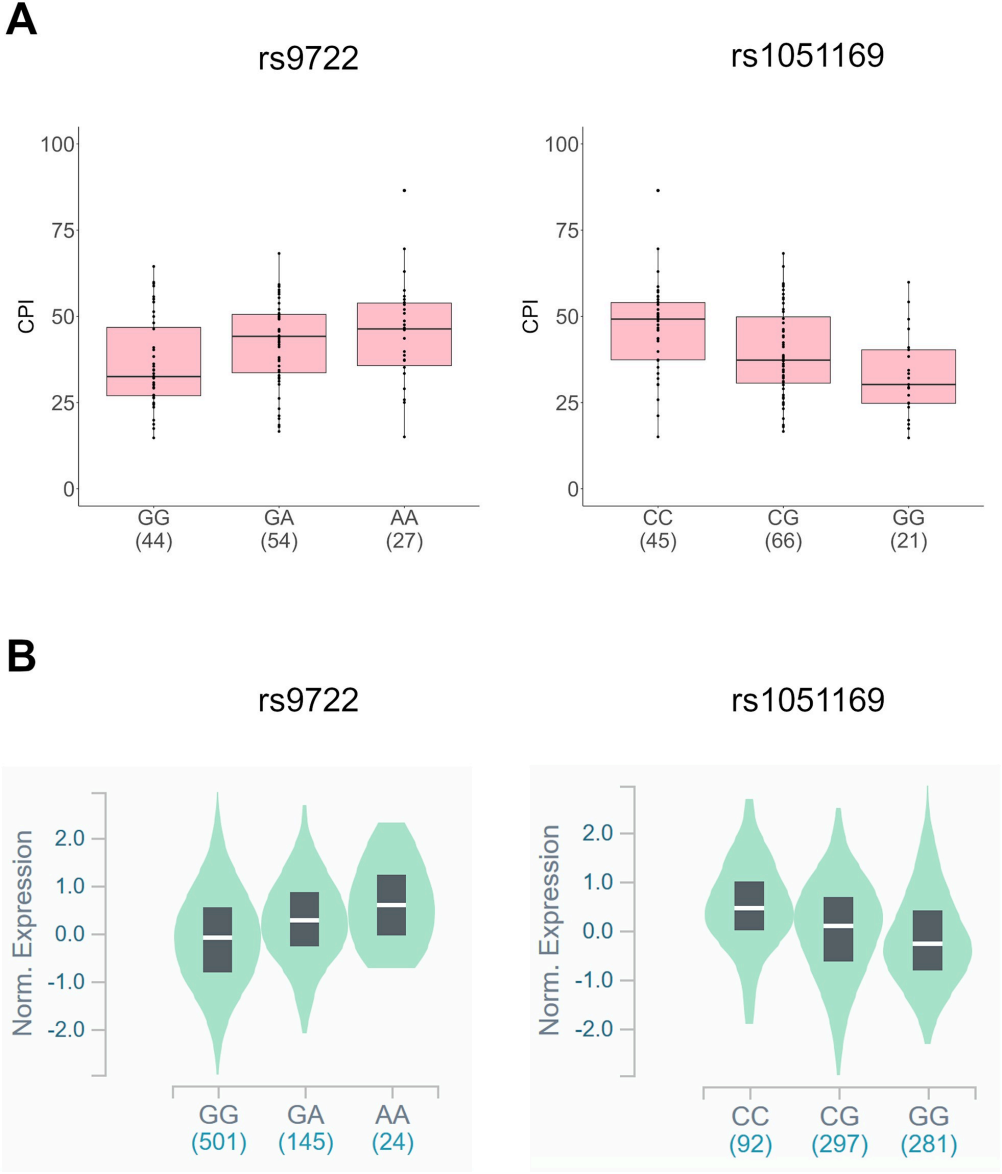
Association of rs9722 and rs1051169 with CPI scores and tissue specific S100B expression. (A) Association of rs9722 and rs1051169 genotypes with CPI scores. (B) Violin plots of single-tissue eQTLs of rs9722 (chr21_46599326_G_A_b38) and rs1051169 (chr21_46602317_C_G_b38) for S100B (ENSG00000160307.9) expression in whole blood from the GTEx Analysis Release V8 (dbGaP Accession phs000424.v8.p2).

We further analyzed GTEx eQTL data to explore any potential association between these SNPs and *S100B* expression. eQTL analysis can be useful in determining functional consequence of genetic variants, particularly that of non-coding sequences. Correlation between tissue specific expression level of genes and genetic variants were obtained from the GTEx database. We found that the A allele of rs9722 associated with a significant increase in S100B expression in whole blood (Normalized effect size = 0.32, p-value = 0.0000042) and the G allele of rs1051169 associated significantly with a decrease in S100B expression in whole blood (Normalized effect size = -0.23, p-value = 0.000006). A graphical comparison of the association of these two SNPs with CPI scores and S100B expression is illustrated in Fig 2.

## Discussion

Genetic polymorphisms in the *S100B* gene have not been previously studied for pain in SCD. Here, we show that *S100B* SNPs rs9722 A allele and rs1051169 G allele may alter baseline pain in SCD even though they were not associated with acute crisis pain. Acute pain in SCD is nociceptive in nature and is a result of tissue damage during vaso-occlusive events, whereas chronic pain is primarily driven by neuropathic mechanisms, thus suggesting the involvement of potentially different molecular pathways [46].

While the association of *S100B* variants, rs9722 and rs1051169, with pain has not been previously evaluated, these variants have been implicated in a number of other CNS conditions. For example, in schizophrenic subjects, the A allele of rs9722 and G allele of rs1051169 were found to be associated with their spatial disability [36, 38]. Another study found that in Han Chinese patients with major depressive disorder (MDD), those carrying the AA genotype of rs9722 had an earlier age of onset than the GA and GG genotypes, and were more vulnerable to recurrent episodes of depression [37]. A similar study in Han Chinese patients with ischemic stroke found that the A allele was associated with increased risk of stroke as well as elevated serum S100B [35].

Studies have also reported altered S100B expression in different pain conditions. In fibromyalgia patients, for example, it was found that increased serum concentrations of S100B were associated with a lower pressure-pain threshold [47]. In migraine patients, serum S100B levels were elevated during a migraine attack as compared to healthy subjects, although maximum concentrations were observed during the pain-free period post migraine attack [48]. Furthermore, S100B expression, used as a biomarker for astrocytic reaction, was found to be upregulated in the spinal dorsal horn of HIV patients with chronic pain as opposed to those without pain [49].

In a recent study by Zhang et al. in sickle cell disease patients, eQTL analysis of *S100B* SNPs revealed that the A allele of rs2154586 was significantly associated with elevated expression of *S100B* and a higher risk of avascular necrosis—a known source of chronic pain in SCD. However, association of *S100B* eQTNs with acute pain episodes did not reach statistical significance after Bonferroni correction [20, 21]. Along the same lines, we found that none of the four S100B SNPs analyzed in our study associated with acute crisis pain, while they associate significantly with chronic pain. Furthermore, Zhang et al. found that serum S100B levels in SCD patients correlated with an increased risk of avascular necrosis, but fewer episodes of acute crisis pain [21], thus highlighting the aforementioned difference between the pathophysiology of chronic and acute pain phenotypes in SCD.

Based on all the prior reports in literature across a spectrum of chronic pain conditions, it could therefore be hypothesized that genetic variants of *S100B* that result in elevated expression of *S100B* may associate with an increased risk of chronic pain in SCD as well. In this study, we found that the A allele of rs9722, which was previously implicated to elevate serum S100B concentration [10], did indeed associate with higher baseline pain. On the other hand, the G allele of rs1051169 associated with significantly lesser pain scores. The same direction of association was also observed in our haplotype analyses. In the haploblock rs9983698 [C>T]—rs9722 [G>A], individuals carrying the risk allele for both the SNPs had significantly higher pain scores than those carrying the common alleles. In the second haploblock rs1051169 [C>G]—rs11911834 [G>T], it was found that the risk allele for rs1051169 alone drove the association with decreased pain scores in our study cohort. Upon analyzing eQTL data from the GTEx database, we found that in agreement with our hypothesis, the A allele of rs9722 associated with increased S100B expression while the G allele of rs1051169 associated with decreased S100B expression in whole blood, corresponding to their respective associations with chronic pain in the same direction, as seen in this study.

It has been well studied that chronic pain is more prevalent in females than males [50]. Influence of sex in pain and analgesia is of much clinical importance [50–52]. Although *S100B* SNPs have not been previously studied in this context, SNPs in several other genes have been reported to exhibit sex-specific associations with pain. For example, studies have reported that OPRM1 A118G has a stronger pain protective effect in males than females [53, 54]. Similarly, a pain sensitive haplotype in the COMT gene was found to have sex specific effect [55]. Sex is also a known influencer of pain in sickle cell [3]. GCH1 SNP rs8007267 served as a fitting example wherein the risk allele associated with painful crisis events and altered endothelial behavior in females but not males [56]. In this study, rs9722 and rs1051169 appeared to influence chronic pain in SCD in a sex-specific manner as well, reaching statistical significance in females but not males. This differential pattern was not only observed in single SNP associations but also in haplotypic associations. These sex specific associations were more pronounced for rs1051169 than it was for rs9722 in our study cohort. It has been previously reported that increase in CSF S100B levels with age is higher in men than women undergoing surgery [57], in contrast to which, a study in migraine patients found that serum S100B was higher in females than males [48].

In summary, our data implicate that *S100B* may play a role in baseline pain sensitivity and heterogeneity in SCD. We have provided evidence that risk allele of SNP rs9722 associate with higher chronic pain scores and that of rs1051169 with decreased pain scores. We also identified two haploblocks in our study cohort and found that specific haplotypes associated with chronic pain. Moreover, single SNP and haplotype associations reached statistical significances in females but not males, suggesting sex-specific interactions. Taken together, our findings suggest a possible role of *S100B* in chronic pain in a sex-specific manner in SCD.

It is also important to note that variability in pain is the result of complex interactions between a multitude of genetic and non-genetic factors [58]. Being a multigenic phenotype, polymorphisms in any single gene can be expected to explain the variability in pain only to a small extent. In that regard, psychosocial factors may also modulate pain behavior and contribute to variability in pain perception [59, 60]. While this makes pain a difficult phenotype to measure and assess, the McGill pain questionnaire used in this study has been well-validated for recording neuropathic pain [26, 29]. This study is limited by the relatively sample size; however, a majority of the study subjects were African Americans with SS-type SCD and the findings present a strong case for chronic pain variability among them. Given the exploratory nature of the current study, larger multisite studies are needed to confirm these findings.

## Supporting information

S1 Appendix (XLSX)
